# Cellular electron tomography of the apical complex in the apicomplexan parasite *Eimeria tenella* shows a highly organised gateway for regulated secretion

**DOI:** 10.1371/journal.ppat.1010666

**Published:** 2022-07-11

**Authors:** Alana Burrell, Virginia Marugan-Hernandez, Richard Wheeler, Flavia Moreira-Leite, David J. P. Ferguson, Fiona M. Tomley, Sue Vaughan

**Affiliations:** 1 The Royal Veterinary College, University of London, North Mymms, United Kingdom; 2 Department of Biological and Medical Sciences, Oxford Brookes University, Oxford, United Kingdom; 3 Peter Medawar Building for Pathogen Research, University of Oxford, Oxford, United Kingdom; University of Melbourne, AUSTRALIA

## Abstract

The apical complex of apicomplexan parasites is essential for host cell invasion and intracellular survival and as the site of regulated exocytosis from specialised secretory organelles called rhoptries and micronemes. Despite its importance, there are few data on the three-dimensional organisation and quantification of these organelles within the apical complex or how they are trafficked to this specialised region of plasma membrane for exocytosis. In coccidian apicomplexans there is an additional tubulin-containing hollow barrel structure, the conoid, which provides a structural gateway for this specialised apical secretion. Using a combination of cellular electron tomography and serial block face-scanning electron microscopy (SBF-SEM) we have reconstructed the entire apical end of *Eimeria tenella* sporozoites; we report a detailed dissection of the three- dimensional organisation of the conoid and show there is high curvature of the tubulin-containing fibres that might be linked to the unusual comma-shaped arrangement of protofilaments. We quantified the number and location of rhoptries and micronemes within cells and show a highly organised gateway for trafficking and docking of rhoptries, micronemes and microtubule-associated vesicles within the conoid around a set of intra-conoidal microtubules. Finally, we provide ultrastructural evidence for fusion of rhoptries directly through the parasite plasma membrane early in infection and the presence of a pore in the parasitophorous vacuole membrane, providing a structural explanation for how rhoptry proteins may be trafficked between the parasite and the host cytoplasm.

## Author summary

Apicomplexan parasites cause a wide range of human and animal diseases. The apical complex is essential for motility, host cell invasion and intracellular survival within a specialised vacuole called the parasitophorous vacuole. We know that molecules important for all these processes are secreted from the apical complex via a set of secretory organelles and there is evidence that some parasite molecules can enter the host cell from the parasitophorous vacuole, but there is little understanding of exactly how this occurs. Here we have used three-dimensional electron microscopy to reconstruct the entire apical end of a coccidian apicomplexan parasite and whole individual parasites. Our results provide important insights into the structural organisation and mechanisms for delivery of parasite molecules via this important area of the cell.

## Introduction

**Fig 1:**
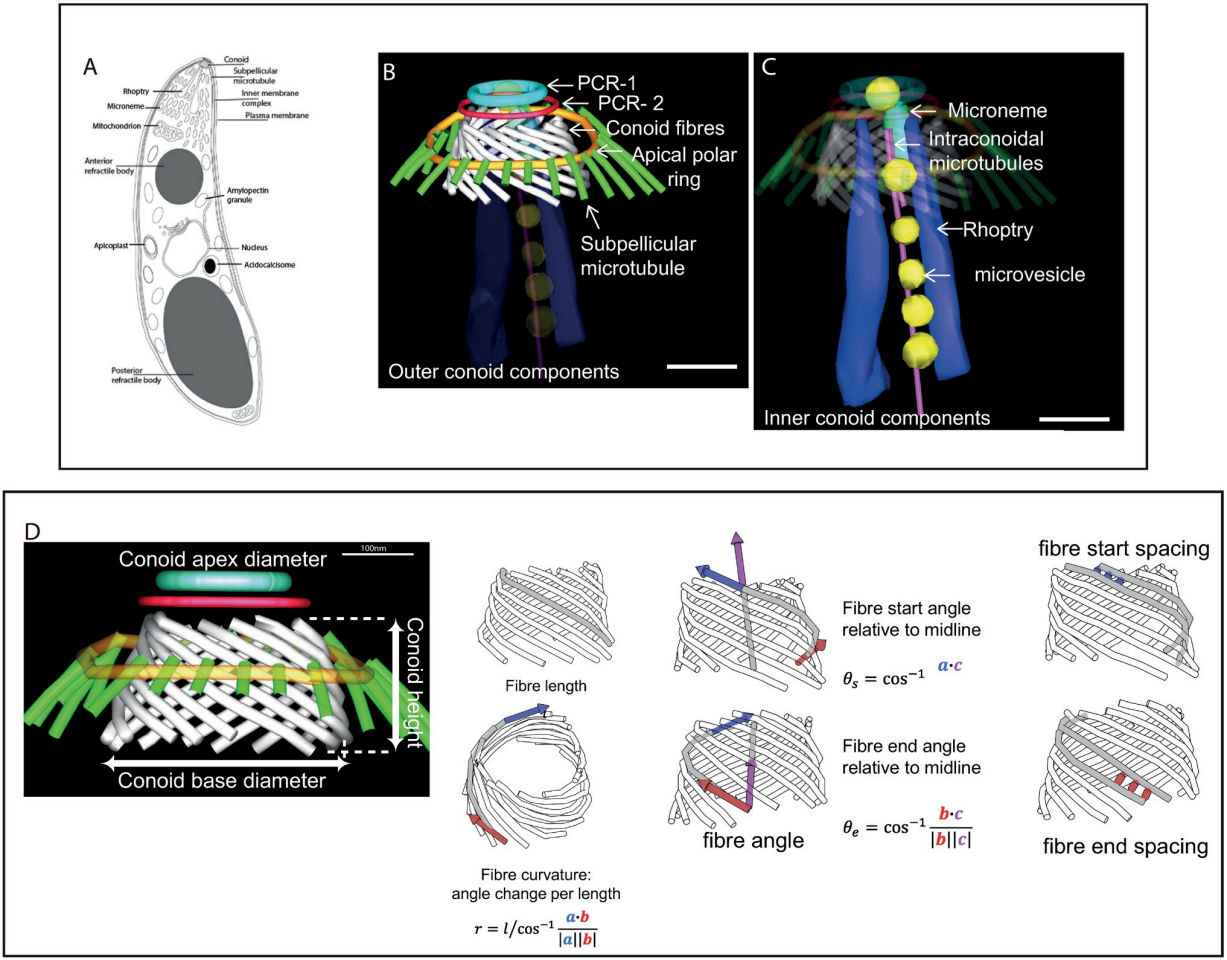
Components of the apical complex in sporozoites of *E*. *tenella*. A: cartoon overview of *Eimeria tenella* ultrastructure. The apical complex is divided into outer conoid (B) and inner conoid (C) components. B and C: Segmentation from a serial section dual axis tomogram. Outer conoid components; conoid fibres (white); 2 pre-conoidal rings (PCR 1 and 2) (light blue and red), apical polar ring (gold) in association with sub-pellicular microtubules (green); B: Inner conoid components, rhoptry (dark blue), microneme (light green), microtubule-associated vesicles (MVs) (yellow), intra-conoidal microtubule pair (pink); Scale bars—200nm; D: Illustrations of measurements carried out of conoid features (n = 14 complete conoid tomograms). Conoid height, apex, and base diameter, fibre length, fibre curvature, fibre angle and fibre spacing. S1 Table contains the measurement data and statistics. Materials and methods describe how the measurements were carried out.

A defining feature of apicomplexan parasites is the apical complex after which the phylum is named. This comprises an apical polar ring to which are attached a set of helically arrayed subpellicular microtubules, and two types of specialised secretory apical organelles called micronemes and rhoptries that are part of the parasite endomembrane system [1] (Fig 1A). The Coccidia sub-class of Apicomplexa, which includes the genera *Eimeria*, *Neospora*, *Sarcocystis* and *Toxoplasma*, also possess a conoid, an apical cone-like hollow structure composed of tubulin-containing fibres [2,3] associated with two pre-conoidal rings and a pair of intra-conoidal microtubules [4]. Transmission electron microscopy analysis in *Toxoplasma gondii* estimated the conoid to contain ~14 tubulin fibres [2], but there was a level of uncertainty in this number due to inherent difficulties in analysing such a complex structure using two-dimensional methods. The two pre-conoidal rings are located at the apical tip of the cell, above the conoid and just under the plasma membrane, and these rings move together with the mobile conoid when it is extended in *T*. *gondii*, although their precise role is not understood [4,5].

Co-ordinated exocytosis of apical secretory organelles is essential for egress, gliding motility, host cell invasion and intracellular survival. Micronemes secrete multiple proteins (MICs) involved in processes such as egress from host cells, parasite motility, cell adhesion and, in conjunction with certain rhoptry proteins, host cell invasion [6–8]. During host cell invasion, a set of rhoptry proteins are secreted into the host cell to facilitate invasion and establish infection [9,10]. Secretion of both rhoptry and microneme proteins occurs from the apical tip and for rhoptry proteins to be inserted into the host cytoplasm there is a requirement to relocate across the rhoptry membrane, parasite plasma membrane and host plasma membrane, but how rhoptry proteins traverse these three membranes is not understood. Freeze fracture analysis of invading tachyzoites of *T*. *gondii* identified a pore-like structure in the parasitophorous vacuole membrane (PVM) overlying the apical end of the parasite [9] that may provide a gateway for secretion. On the surface of the parasite membrane an apical rosette of membranous particles is embedded overlying the apical end [10,11] and recent cryo-EM studies have demonstrated an interaction with the apical rosette, an apical vesicle and individual rhoptries that could be important for rhoptry secretion [11,12]. Finally, rhoptries are occasionally seen as empty sacs during host cell invasion, which are proposed to represent discharged rhoptries [9,13]. Taken together, these ultrastructural studies are beginning to reveal mechanisms for rhoptry secretion.

Rhoptries have been visualised within the conoid in many studies [14], but there has been some uncertainty over the precise location of micronemes either within the conoid [1] or at the base of the conoid when it is protruded [15]. Here, we used high resolution cellular electron tomography to reconstruct the apical complex of *Eimeria tenella* sporozoites. Data from serial electron microscopy tomography and serial block-face scanning electron microscopy (SBF-SEM) were combined to produce 3D models showing how secretory organelles are arranged for exocytosis. Our data provide evidence of a role for the intra-conoidal microtubules in secretory organelle organisation into the conoid and successive secretion of multiple micronemes. In addition, we identified fusion of a rhoptry with the parasite membrane overlying the conoid with a pore in the parasitophorous vacuole membrane that may be important in the delivery of rhoptry contents to the host cytosol early in infection.

## Results

### Cellular electron tomography of the apical complex reveals a highly ordered gateway for secretion

To investigate the detailed ultrastructure of the conoid and how the secretory organelles are organised in the apical complex, serial section electron tomography was performed on freshly hatched extracellular sporozoites and intracellular/invading sporozoites (N = 17, serial section dual axis tomograms). These tomograms encompassed the entire apical end covering ~2.5 μm^3^. Segmentation of the conoid and associated structures was carried out for each serial tomogram (e.g. S1 Movie and selected slices through a serial section tomogram images in S1 Fig). Due to the complexity of the overall structure, we divided the apical complex into two parts: the structures/organelles that surround the conoid barrel are referred to as the ‘outer’ conoid components (Fig 1B) and the structures/organelles contained within the barrel of the conoid are called ‘inner’ conoid components (Fig 1C). The outer apical complex includes an electron-opaque apical polar ring (APR) (Fig 1B, gold). Our work confirms there are 24 evenly spaced subpellicular microtubules (Fig 1B; green) radiating from the APR and extending towards the posterior of the cell, as identified in all 17 tomograms. Two ring-shaped structures distal to the conoid—known as pre-conoidal ring-1 (PCR-1) (Fig 1B, light blue) and pre-conoidal ring 2 (PCR-2) (Fig 1B, red)—complete the outer conoid components. The outer apical complex is enclosed by the plasmalemma and partially enclosed by the inner membrane complex (IMC), which is interrupted apically to form a circular apical opening (not shown in the tomograms). This is located distal to the apical polar ring (APR).

The inner apical complex components include a pair of centrally located intra-conoidal microtubules (Fig 1C, pink). We discovered that one of these microtubules starts and terminates in line with the height of the conoid, whilst the other extends into the cell interior past the base of the conoid and beyond the region covered in our tomograms, which has not been reported previously (Fig 1C, pink, S1 Fig: slices 10 and 11, arrow in slice 10). Microtubule-associated vesicles (MVs) of varying number (see below) are closely associated with the two intra-conoidal microtubules and appear as a row of electron-opaque spheres leading posteriorly from each conoid (Fig 1C, yellow; S1 Fig: slice 11, asterisks). MVs have been identified in this configuration in numerous studies, but their composition is unknown [12,15]. Our tomograms clearly show both rhoptries (Fig 1B and 1C, dark blue; S1 Fig: slices 7–8, “R”) and micronemes (Fig 1C, light green; S1 Fig: slice 10, “M”) located within the conoid area of all 17 tomograms in *E*. *tenella* (see below).

When viewed as a three-dimensional reconstruction, the conoid appears as an open truncated cone formed from closely apposed tubulin-containing helical fibres [2] which follow a left-handed helical path towards the apical end of the parasite (Fig 1B, white). Negatively stained whole-mount cytoskeletons of *T*. *gondii* estimate that the conoid is composed of 14–15 helically arranged tubulin-containing fibres [2,16], but high resolution tomography of the conoid had not been carried out to confirm this. Our high-resolution tomograms show that there are between 13 and 16 fibres in the conoid, and there can be variation in fibre number even in genetically identical sporozoites within the same sporocyst [16] (S2A Fig). Detailed quantification of conoid height, diameter, fibre angle, fibre length and fibre spacing was carried out on 14 of the 17 reconstructed tomograms where an uninterrupted view of the fibres across each serial dual axis tomogram was possible using iMOD (see Materials and methods) (Fig 1D, S1 Table). Average height of the conoid was 193 nm (+/- 28 nm SD) and the diameter at the base of the conoid (average = 331 nm +/- 19 nm SD) was greater than the apex (average = 252 nm +/- 19 nm SD), illustrating the open cone organisation (Fig 1D, S1 Table). Average conoid fibre length was 422 nm (+/- 40 nm SD) and the average radius of curvature for conoid fibres was 448 nm (+/- 51 nm SD) (Fig 1I, S1 Table). The fibre angle relative to the midline of the conoid was calculated and there was only a small change up of 0.8° from an average of 50.9° (+/- 0.8° SD) to 51.7° (+/- 1° SD) (*p* = 3.1×10^−2^, paired T-test) from the start of the conoid fibres to the base of the conoid fibres (Fig 1D). Fibre spacing was determined by measuring 10 locations along each fibre to the adjacent fibre (see Materials and methods). There was an increase in spacing between fibre start (apex of conoid) and end (base of conoid) in each conoid up by 6.0 nm from an average of 28.3 nm (+/- 3.2 nm) to 34.3 nm (+/- 3.6 nm) (*p* = 8.2×10^−6^ paired T-test) (Fig 1D), suggesting that the increase in spacing and change in fibre angle play a role in providing the increasing diameter of the conoid from apex to base.

### Micronemes, rhoptries and microtubule-associated vesicles are organised along the intra-conoidal microtubules and closely associated with the plasma membrane overlying the conoid

**Fig 2:**
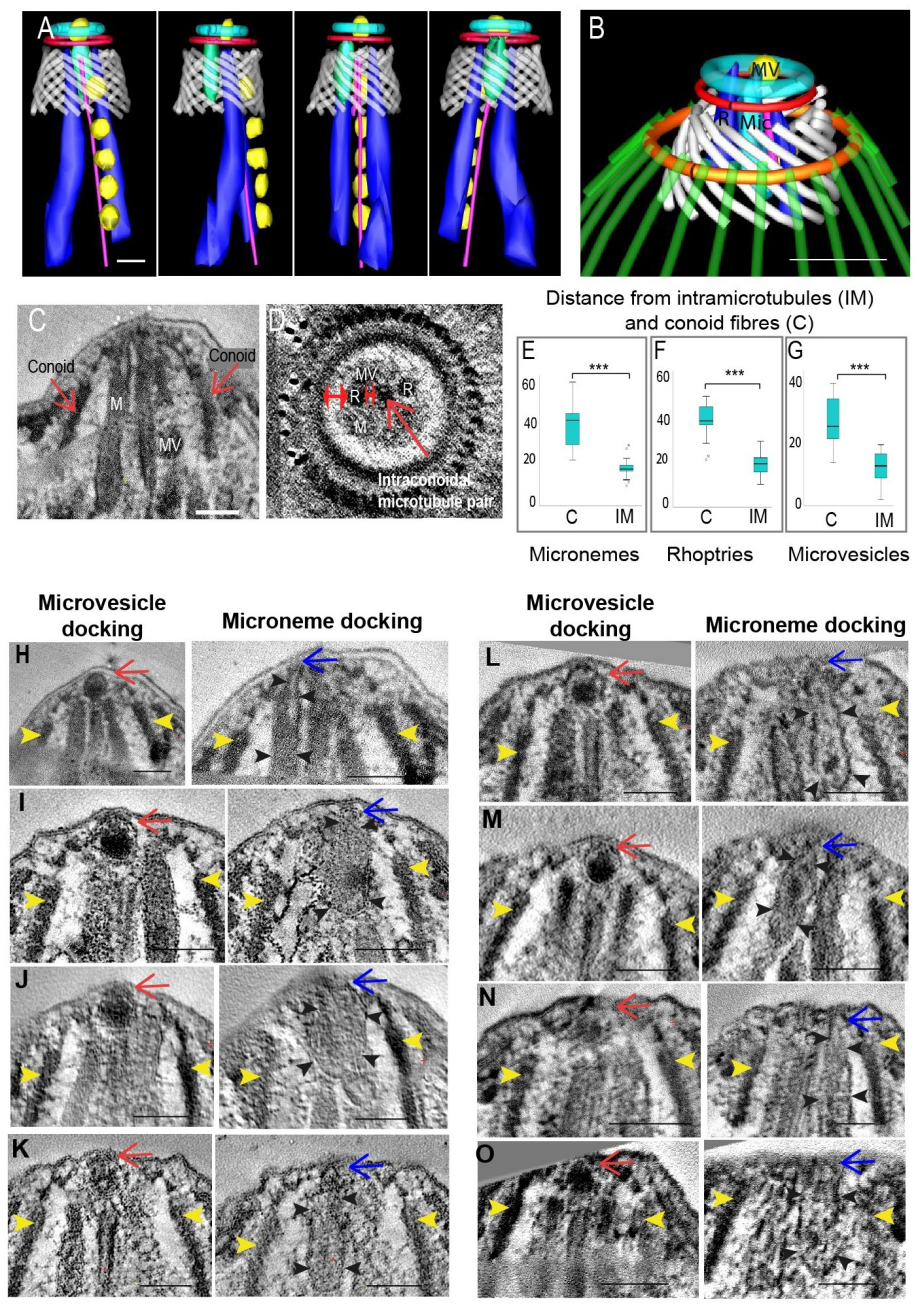
Secretory organelles and their association with the intra-conoidal microtubules and overlying the plasma membrane. A: Series of rotational views of a segmentation created from one serial section dual axis tomogram illustrating the spatial grouping and alignment of microneme (light green), rhoptries (dark blue) and MVs (yellow) with the intra-conoidal microtubule pair (pink); B: Segmentation of a serial section dual axis tomogram illustrating the relative positioning of a microneme (light green), rhoptry (dark blue) and MVs (yellow) within the conoid area of the parasite; C and D: slices taken from different tomograms (C) longitudinal view and (D) cross section view illustrating how measurements were taken showing the distances from a microneme (M), rhoptry (R) and microtubule-associated vesicle (MV) to either the intra-conoidal microtubules or conoid. Double-headed red arrows in (D) show where the measurements were taken, single arrow in C for the identification of the conoid in longitudinal sections. Single arrow in D is the location of intra-conoidal microtubules; E-G: Micronemes, MVs and rhoptries were significantly closer to the intra-conoidal microtubules (IM) than to the conoid (C) (t-test, p < 0.0001) (N = 17); H—I: Longitudinal slice views from 8 serial section tomograms illustrating a MV (red arrows), microneme (blue arrows and black arrowheads) in close association with the plasma membrane overlying the conoid in each tomogram. Yellow arrowheads show the outer edge of the conoid in each example. Scale bars– 100nm.

The serial tomograms generated encompassed the full area of the conoid at sufficient resolution to allow us to establish the precise location and number of each type of secretory organelle. Although microneme proteins are known to be secreted at the apical pole, the precise location and mechanism of trafficking of microneme proteins into the apical complex is uncertain [1,15]. In our dataset we discovered 1–2 rhoptries, 1–5 micronemes and 1–3 MVs within the barrel of the conoid in all tomograms (Fig 2A–2D and S3 Fig). S3 Fig shows examples of 5 freshly excysted sporozoites with differing number of micronemes within the conoid area as well as those entering the conoid area. Rhoptries, micronemes and microtubule-associated vesicles (MVs) were always closely associated with intra-conoidal microtubules, which are located centrally within the barrel of the conoid (Figs 1C, 2A and 2B and S1 Movie). To quantify the spatial relationships between the intra-conoidal microtubule pair and micronemes, rhoptries and MVs, the distances between each organelle and the intra-conoidal microtubules was measured, and between each organelle and the conoid fibres (Fig 2C–2G). One of each organelle type (situated within the conoid) was chosen in each tomogram and measurements were taken of the shortest distance between organelle and conoid/microtubule, as visually observed. These measurements reveal that micronemes, rhoptries and MVs are located significantly closer to the intra-conoidal microtubule pair within the central barrel of the conoid than to the fibres of the conoid barrel (Fig 2E–2G), suggesting a potential role for the intra-conoidal microtubules in organising the secretory organelles within the conoid in readiness for secretion.

The plasma membrane overlying the conoid was checked for evidence of fusion events or close apposition with microneme, rhoptry or spherical vesicle membranes. All rhoptries within the conoid were in close apposition with the plasma membrane, but fusion with the plasma membrane was not observed. MVs were organised in single file along the intra-conoidal microtubules as shown in *T*. *gondii* and *C*. *parvum* [12] and there were always 1 or 2 micronemes and at least 1 MV located in close apposition or in contact with the plasma membrane overlying the conoid (Fig 2H–2O). This suggests a sequential trafficking of micronemes and MVs along the intra-conoidal microtubules to the plasma membrane overlying the conoid, in preparation for secretion. Recent cryo-EM work with *T*. *gondii* and *C*. *parvum* showed a vesicle (called an apical vesicle) that is a similar in size to MVs providing a link between a rhoptry and the plasma membrane overlying the conoid, which is proposed to be important for rhoptry discharge [12]. One vesicle that we identify as a MV was always located beyond the end of the intra-conoidal microtubules and closely associated with the plasma membrane in all our tomograms (Fig 2A, 2B and 2H–2O). This apical MV was clearly associated with a rhoptry in 2 tomograms, but not clearly associated with a rhoptry in all of our other freshly excysted sporozoite tomograms, which could further point to a possible association with rhoptry secretion that only occurs during invasion.

**Fig 3:**
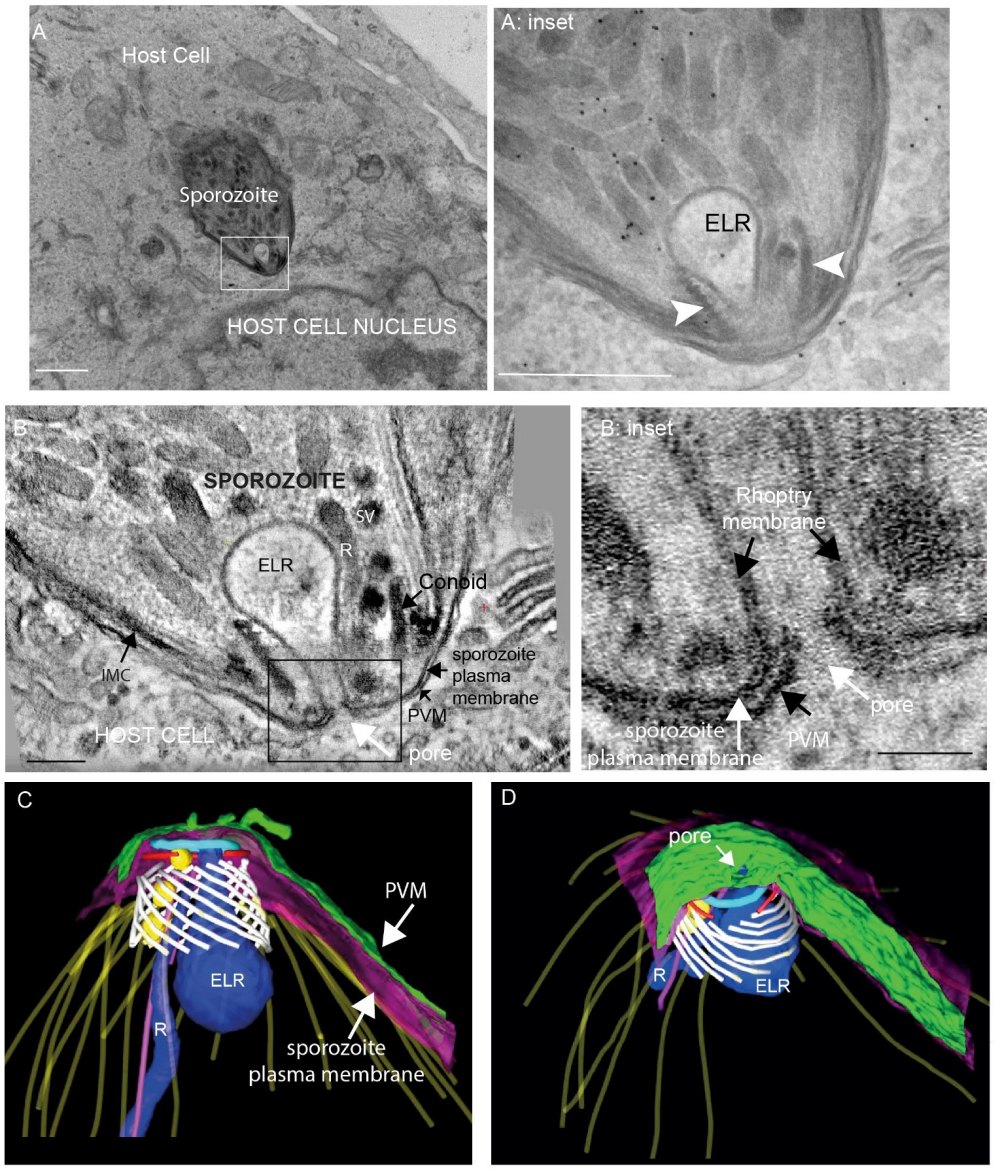
Characterisation of an electron lucent rhoptry in an intracellular or invading sporozoite. A: Slice from a Transmission electron microscopy image illustrating an invading or intracellular sporozoite with an electron lucent rhoptry (ELR) (box and A: inset) close to a host cell nucleus (30 min post-infection sample) scale bar 1μm; A: inset: Higher magnification of A illustrating the ELR within the conoid (white arrowheads). Scale bar 500nm; B and B: inset: Slice from a tomogram showing the electron lucent ‘empty’ rhoptry (ELR) which appeared to be continuous with the parasite plasma membrane and associated at its apex with a pore in the parasitophorous membrane creating a channel passing through both the parasite plasma membrane and the PVM connecting the ‘empty rhoptry’ directly with host cell cytoplasm. B–scale bar 100nm; B inset scale bar 50nm; C and D: Segmentation of a serial tomogram outlined in A and B to illustrate the three dimensional organisation and relative positioning of the electron lucent rhoptry (ELR–dark blue), electron dense rhoptry (R–dark blue), conoid fibres (white), MVs (yellow), parasite plasma membrane (purple), parasitophorous vacuole membrane (green), PCR-1 and 2 (light blue and red), subpellicular microtubules (yellow), intra-conoidal microtubules (pink).

Our dataset included two serial section dual-axis tomograms of the apical end of sporozoites either fully or partially invading Madin-Darby bovine kidney cells (MDBK). In one of these sporozoite tomograms fixed at 30 min post-infection, a rhoptry was identified with an electron-lucent interior instead of the usual electron dense rhoptry organisation (Fig 3A and 3A: inset, 3B and 3B: inset and S2 Movie). The electron-lucent rhoptry (ELR) differed in shape from the electron dense rhoptry (R) in the same and other tomograms, having a broader base and flask-shape (Fig 3C; ELR) and this phenomenon has been noted in earlier ultrastructural studies [9,17]. Intriguingly, the ELR appeared to be continuous with the parasite plasma membrane as if the rhoptry had fused with the parasite plasma membrane overlying the conoid, to release its contents (Fig 3B and 3B: inset). Freeze-facture images of invading *T*. *gondii* cells identified a distinct pore-like structure in the plasma membranes of the parasite [9] and in our tomograms we identify this as the point of rhoptry fusion where a hole in the PV membrane (PVM) appears to create a channel passing through both the parasite plasma membrane and the PVM, connecting the electron-lucent rhoptry directly with host cell cytoplasm (Fig 3B and 3B: inset, 3C and 3D and S2 Movie). Notably, there was also an electron dense elongated rhoptry within the conoid of the same parasite, suggesting the possibility of sequential rhoptry discharge, but how this is orchestrated is not currently understood. Overall, our extensive tomography analysis of both freshly excysted and invaded sporozoites revealed that different types of secretory organelles converge within the conoid and are closely associated with the intra-conoidal microtubules, forming what appears to be a highly ordered secretory gateway at the apical end of the cell.

### Whole cell reconstructions of individual sporozoites reveals the organisation and location of major sporozoite organelles

**Fig 4:**
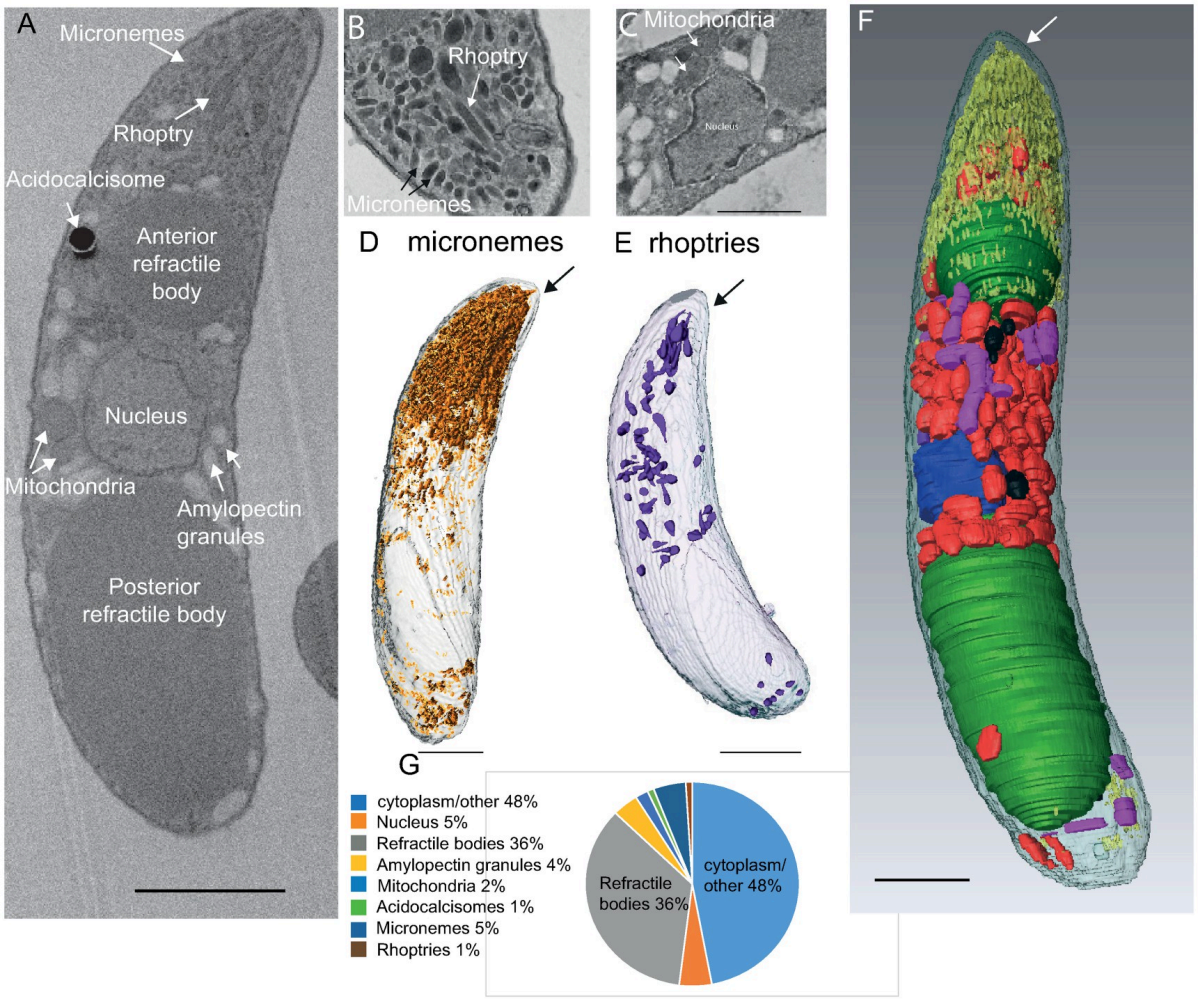
SBF-SEM quantification of micronemes, rhoptries and other major organelles in freshly excysted sporozoites. A: Longitudinal section slice from an SBF-SEM dataset illustrating the major organelles. B and C: Thin section TEM images to illustrate identification of major organelles. D and E: Quantification and location of microneme and rhoptry organelles in SBF-SEM whole cell reconstructions. F. Combined segmentation illustrating the positioning of all the major organelles: micronemes–yellow, refractile bodies–green, amylopectin granules–red, acidocalcisomes–black, rhoptries–purple, nucleus–blue—Scale bars– 1μm; G. Relative volumes of major organelles in freshly excysted sporozoites.

Our tomography data revealed that only a limited number of individual secretory organelles are located within the conoid area, yet many studies show the presence of hundreds of micronemes within the apical end of the parasite outside the conoid area. To better understand the broad localisation and number of secretory organelles, freshly excysted sporozoites were prepared for serial block face-scanning electron microscopy (SBF-SEM), which allows automated collection of datasets containing hundreds of sequential serial sections. A total of 25 whole individual sporozoite cells were identified in the aligned SBF-SEM series which were reconstructed and analysed for organelle number and three-dimensional organisation. A single slice of a freshly excysted sporozoite illustrates that most major organelles were visible by SBF-SEM (Fig 4A) and compares with thin section transmission electron microscopy images (Fig 4B and 4C). Segmentation in 4F illustrates the overall three-dimensional organisation. S3 Movie illustrates a portion of the SBF-SEM dataset showing 25 slices containing a sporozoite. Organelle volume and number were determined by manual segmentation of each organelle, and the relative abundance and location of all identifiable organelles (including micronemes and rhoptries) were analysed (Figs 4F and S4). Due to the number and close packing of micronemes located mainly at the apical end of the sporozoite, it was not possible to accurately count them. Instead, micronemes were segmented using a combination of pixel-density thresholding and manual area selection (Fig 4D, n = 4). The total mean whole cell volume of individual sporozoites was 61.27 μm^3^ (S4A Fig) with micronemes comprising 5% of whole cell volume and rhoptries <0.5% of whole cell volume (Fig 4G). This quantitative analysis revealed that refractile bodies make up 36% of whole cell volume (2 per cell in freshly excysted sporozoites), with the nucleus (1 per cell) and amylopectin granules (~196/cell) each comprising 4%, mitochondria (~14/cell) 2% and acidocalcisomes (~13/cell) 1% of whole cell volume (S4 Fig for full quantification). The majority of micronemes were densely packed at the apical end of the sporozoite (Fig 4D). Rhoptries were observed as club-shaped structures with a rounded ‘bulb’ region and an elongated ‘neck’ region. They were also mostly found towards the sporozoite apical end, although some rhoptries were present in the central and posterior parts of the cell (Fig 4E). Unfortunately, the MVs observed by tomography (in association with the intra-conoidal microtubules, e.g Fig 1C) were not clearly identified in the reconstructed whole cell SBF-SEM data. By combining findings from tomography and SBF-SEM, we conclude that *E*. *tenella* sporozoites contain an abundance of micronemes and rhoptries, and only a small number of these are seen within the conoid where secretion occurs, suggesting that there is significant directed movement of these secretory organelles converging at the conoid.

## Discussion

Secretion of microneme and rhoptry contents from the apical end of apicomplexan zoites is well documented and hundreds of micronemes have been visualised closely packed at the apical end of many different apicomplexan parasites by classical thin section transmission electron microscopy (TEM) thin sections (for review see [1]). Our detailed three-dimension reconstructions now highlight the large number of densely packed apical micronemes in *E*. *tenella* sporozoites compared to the relatively low numbers present in the tachyzoites, although the reason for this difference is unclear. Exactly how individual micronemes are trafficked from this closely packed area into the conoid for secretion from this small portion of plasma membrane is not well understood. In *T*. *gondii*, focussed ion beam scanning electron microscopy (FIB-SEM) images did not find micronemes within the conoid area and it was proposed that microneme secretion could occur adjacent to the conoid, close to the APR and the subpellicular microtubules, where a membrane space could open up as the conoid protrudes [15], rather than secretion occurring at the plasma membrane directly overlying the conoid. However, further studies using the same technique in *T*. *gondii* did observe a microneme inside the conoid area and cryo-EM studies in *T*. *gondii* and *C*. *parvum* also show micronemes within the conoid area [11,12,18]. Intra-conoidal microtubules have been proposed to be involved in secretory cargo trafficking [19] and cryo-EM analysed the shape and number of MVs lined up along the these two microtubules. We also show MVs along these microtubules and that micronemes and rhoptries are also closely associated. One intra-conoidal microtubule terminates at the base of the conoid, but the other is longer, extending into the cell. This longer microtubule could be required for guiding/trafficking of micronemes and MVs into the conoid space, where it is ideally positioned to interact with secretory organelles outside the conoid area and ‘guide’ them to the conoid for discharge. We propose a role for intra-conoidal microtubules in the trafficking of individual micronemes and MVs through the conoid, and the co-ordination of an orderly system for transporting these organelles to the membrane in preparation for docking, fusion, and release of contents. This suggests that the intra-conoidal microtubules are a major organiser of trafficking within the conoid area. This would be consistent with the observation that TgDCL8a (component of the microtubular motor complex) affect micronemes transport in *T*. *gondii* [20] (Lentini et al., 2019).

Careful analysis of tomograms did not find direct evidence for fusion of microneme or MV membranes with the plasma membrane overlying the conoid, despite their close proximity to the plasma membrane in nearly all tomograms. This might be due to the fixation method used in this study or could indicate that the process is very fast and therefore extremely difficult to capture in still images, but certainly the signalling cascade for membrane fusion exists in apicomplexan organisms (for review see [1]. Further work using high pressure freezing or cryo-EM may be required to capture these events clearly. A combination of cryo-EM and freeze fracture studies using isolated *T*. *gondii* tachyzoites revealed rhoptry exocytosis is dependent on a rosette of intramembrane particles that are embedded in the parasite plasma membrane overlying the conoid [9] with an apical vesicle forming a connection between both the intramembranous rosette and a rhoptry [11,12]. It is likely that the events captured by cryo-EM in these studies may represent the discovery of intermediate steps in rhoptry secretion in these isolated tachyzoites pre-invasion. We were able to identify the close apposition of the most apical MV with a rhoptry in 2 freshly excysted tomograms, but not in the other tomograms. A previous study of an invading tachyzoite, using freeze fracture imaging identified a 40nm pore between the parasite and host membranes that was speculated to be involved in rhoptry secretion directly into host cells [9]. Our data supports this hypothesis, and further shows this pore linking an electron-lucent rhoptry through the parasite membrane into the host cell cytosol, suggesting that a rhoptry fusion event can occur between the three membranes of the rhoptry, parasite and host cell membrane during invasion. Most ultrastructural imaging of rhoptries are of electron-dense, elongated club-shaped organelles, but occasionally shorter flasked-shaped electron-lucent rhoptries have been noted in invading parasites and these are proposed to represent empty rhoptries after discharge of contents into the host cytoplasm [9,17]. Many apicomplexan parasites have multiple rhoptries located at the apical complex and there are still outstanding questions on the mechanism of successive rhoptry fusion and what happens to discharged rhoptries. It is probable that rhoptry fusion is a highly dynamic event that takes place only at specific stages during parasite/host cell interactions, which might explain why they have been visualised only on rare occasions. In addition, the pore is small (~40nm), which would make it even more difficult to obtain clear images by thin section transmission electron microscopy.

Our detailed measurements and quantification of conoid contents shows a highly ordered organisation of conoid fibres. Importantly, the measurements support a model where cell-to-cell heterogeneity in fibre number occurs even between individual sporozoites at different locations (within oocysts, free freshly-excysted or intracellular prior to trophozoite development). Our conoid fibre measurements revealed a high curvature and supports the hypothesis that the unusual comma-shaped arrangement of the tubulin-containing protofilaments of the conoid fibres are linked to differences in curvature found in conventional microtubule protofilaments [2]. Intriguingly in all parasites there were always 24 subpellicular microtubules on the APR, which presumably places a physical constraint for the minimum and maximum possible diameter of the APR and its associated structures and thus, dictates the minimum and maximum overall dimensions of the apical complex at least in the sporozoite stage of this parasite.

Combining high resolution cellular electron tomography and lower resolution SBF-SEM data is a powerful way of combining investigations at high resolution of specific areas of a cell with a whole cell view. These datasets reveal a highly organised gateway for trafficking of secretory organelles to the conoid area of the apical complex. Further work will be required to understand the role of rhoptry fusion and pore formation during invasion and how individual micronemes are trafficked into the conoid area from such a large cluster underlying the conoid area.

## Methods

### Ethics statement

The studies described here were carried out in strict accordance with the Animals (Scientific Procedures) Act 1986, an Act of Parliament of the United Kingdom. All sample collection, animal studies, and protocols were approved by the Royal Veterinary College (UK) Animal Welfare and Ethical Review Board (AWERB) and the United Kingdom Government Home Office.

### Infection of chickens and sporozoite purification

Three-week-old Lohmann chickens (purchased from APHA Weybridge) kept under specific pathogen free conditions were orally infected with 4,000 sporulated *E*. *tenella* Wisconsin strain oocysts (Shirley, 1995). Oocysts were harvested at 7 days post-infection and excystation and sporozoite purification performed as previously described (Pastor-Fernández et al., 2020).

### In vitro *E*. *tenella* infections

The NBL-1 line of MDBK cells (ECACC-Sigma-Aldrich, Salisbury, UK) were prepared as previously described (Marugan-Hernandez et al; 2020). One millilitre of cell-culture medium containing 0.35 x 10^6^ MDBK cells was added to wells of a 24 well culture plate. Cells were left to settle for up to 3 hr at 38°C, 5% CO_2_, prior to infection with sporozoites. Freshly-purified sporozoites were pelleted by centrifugation at ~ 600 RCF for 10 min and re-suspended in cell-culture medium at a concentration of 3.5 million sporozoites per ml. One millilitre of sporozoite suspension was added to each MDBK-cell containing well.

### Preparation of freshly excysted sporozoites for electron microscopy

Freshly purified sporozoites (~10–50 million) were suspended in 1 ml of primary fix (2% freshly prepared formaldehyde solution (Sigma-Aldrich), 2.5% electron microscopy grade glutaraldehyde (TAAB) and 0.1 M sodium cacodylate buffer (TAAB) in double distilled (dd H_2_O)). Sporozoites were left in primary fixative for two hours at 4°C. Fixed sporozoites were washed five times in 0.1 M cacodylate buffer pH 7.4 for 10 min. Sporozoites were pelleted by centrifugation and incubated in 2% osmium tetroxide (TAAB) in 0.1 M cacodylate buffer for 60 min at 4°C. For uranyl acetate staining, sporozoites were added to 1 ml molten 3% agarose (2-Hydroxyethyl agarose–Sigma-Aldrich, dissolved in ddH_2_O), centrifuged and incubated at 4°C for 5 min. Approximately 1 mm^3^ blocks were cut from the part of the agarose containing sporozoites and incubated in freshly-filtered 2% aqueous uranyl acetate (Agar scientific) in the dark at 4°C overnight. Sporozoites were washed in ddH20 and then dehydrated by a series of 20 min incubations in acetone/ddH_2_0 solutions. Samples were incubated for 2 hours in 25% epoxy resin (TAAB 812 resin premix kit) in acetone; overnight in 50% resin in acetone; 6 hrs in 75% resin in acetone; overnight in 100% resin, and finally, two changes of 100% resin for 2 hours each. Polymerisation was achieved by incubation at 60°C for 24 h.

### Preparation of host cells infected with sporozoites for electron microscopy

Electron microscopy grade glutaraldehyde was added to the infected MDBK cells for 15 min followed by fixation as above. After dehydration, propylene oxide was added to the culture wells and the media containing the cells were centrifuged to form a pellet, then embedding continued as above.

### Electron tomography and measurements

Transmission electron tomography was performed using one of several transmission electron microscopes: H-7560 (Hitachi), Spirit (Tecnai, FEI/Thermo Fisher Scientific) or Talos (FEI/Thermo Fisher Scientific). Sections were cut at 150 nm thickness for 120 kilovolts (kV) (Hitachi H-7560 or Tecnai Spirit electron microscopes) and at 150 nm-200 nm thickness for 200 kV (FEI Talos electron microscope). Tomogram tilt series generated using the Hitachi H-7560 electron microscope were taken from -60° to +60° with intervals of +1°. For tomography data acquisition using the Tecnai Spirit electron microscope, automated centring, focus adjustment, tilt setting, and image capture were performed using Xplore*3D* software by FEI. For tomography data acquisition using the FEI Talos electron microscope, automated centring, focus adjustment, tilt setting, and image capture were performed using DigitalMicrograph (Gatan) with SerialEM plug-in (Mastronarde, October 2005). Regardless of the microscope used, dual axis tomograms were collected by rotating the grid by 90° and repeating the tilt series image collection. Tomogram image data series processing, segmentation of the tomograms (modelling) to produce three dimensional reconstructions and all measurements from the tomograms were also carried out using IMOD software (Kremer et al., 1996) (University of Colorado, Boulder). Measurements of distance from intraconoidal microtubules were carried out in IMOD. Custom scripts were used to determine quantitative measures of conoid fibre properties illustrated in Fig 1D. Three of the 17 tomograms were excluded from the detailed conoid morphometric analysis so that only fibres where the start and end were within the imaging volume were included. The conoid 3dmod files were represented as contours (multi-segment lines defined by a series of 3D coordinates) within a single object, with contours oriented with their starts at the conoid wide end and running towards the narrow end, numbered in clockwise order looking at the conoid wide end. Fibre length was measured from the sum line segment lengths in the contour. Fibre curvature was measured as the angle between the vector defined by the first two and last two points of the contour, divided by fibre length. To determine fibre spacing, contours were sampled at 10 locations along each line segment and determined the distance to the nearest point in the previous (next anticlockwise) or next (next clockwise) contour. Mean spacing between fibres at the narrow and wide end of the conoid were taken as the average of points sampled in the end 70nm of the contour measured to the previous contour and the start 70nm of the contour measured to the next contour respectively. To determine fibre angle relative to the orientation of the conoid the conoid midline was determined by taking the average of all contour start points and end points to define the midline start and end respectively. Then, mean line segment angle relative to this midline at the narrow and wide end of the conoid were taken as the average of angle measurements for line segments wholly or partially within 70nm of the contour start or 70nm of the contour end respectively. Paired T-tests were used to compare fibre spacing and angle at the wide end of each conoid in comparison to its narrow end, using the average of all fibres in each conoid.

### Serial block face scanning electron microscopy (SBF-SEM) image acquisition

SBF-SEM data was collected using a Zeiss Merlin scanning electron microscope with Gatan 3View automated sectioning and image capture system. Samples were trimmed to ~1 mm^3^ and mounted on 3view sample pins using an epoxy conductive adhesive from Circuitworks. After insertion of the mounted block, the intra-microscope diamond-knife was advanced towards the block-face by 200 nm cutting-strokes until sectioning of the block face was observed. The block-face was then imaged using a scanning electron beam with 3–5 kV accelerating voltage. Electron signal was detected using a back-scatter electron detector (OnPoint, Gatan) and nitrogen gas was injected to raise chamber pressure to 30pa. **S**BF-SEM data was processed using IMOD software. (University of Colorado, Boulder) and run through Cygwin command line interface. Segmentation was also carried out using AMIRA software.

### Statistical analysis

Statistical analyses were performed using IBM SPSS version 25 software. A t-test was used to test for an association between a continuous variable and a binary categorical variable where there was normal distribution for both groups.

## Supporting information

S1 Fig Series of 15 tomographic slices through a representative tomogram, conoid–yellow arrowheads, micronemes–arrows throughout the series, microneme within the conoid (10,11—M), Rhoptry (7, 8—R), long and short intra-conoidal microtubules (10—arrow), MVs (11—Asterisks), conoid fibres and inset (13). Scale bar—500nm.
(TIF)

S2 Fig Conoid fibre number variation in sporozoites from pre-excystation sporocysts, freshly excysted and intracellular. A: Segmentation of sporozoite conoids from pre-excystation sporozoites within sporocysts, freshly excysted and intracellular sporozoites. The numbers next to sporocysts indicates matching sporozoites within a sporocyst;.
(TIF)

S3 Fig A selected tomogram slices from 5 (A-E) serial tomograms to illustrate the number of micronemes (mic) and rhoptries in the conoid area. All micronemes that were either partially or fully within the conoid were included. Conoid is highlighted with yellow arrowheads in all examples. Tomogram A illustrates the presence of 2 rhoptries and 5 micronemes within the conoid. Mic 1 and 3 are closest to the plasma membrane overlying the conoid. Mic 2, 4 and 5 have partially entered the conoid area; Tomogram B, slices from a tomogram containing 2 rhoptries and 3 micronemes. Mic 1 is closest to the plasma membrane; Tomogram C shows slices from a tomogram with 2 rhoptries and 2 micronemes; Tomogram D illustrates slices from a tomogram containing 2 rhopries and 2 micronemes. Mic 1 is closest to the plasma membrane; Tomogram E, slices from a tomogram containing 2 rhoptries and 2 micronemes.
(TIF)

S4 Fig Volume and numbers of major organelles from whole cell reconstructions of freshly excysted sporozoites by SBF-SEM. Analyses was calculated from segmented SBF-SEM data for each organelle in freshly excysted sporozoites. For each organelle the mean volume of an individual organelle is included, SD = standard deviation, COV = co-efficient of variation, range of volumes of a particular organelle or cell volume. The number of organelles per cell is included for amylopectin granules, acidocalcisomes and mitochondria. AP = Apical end of the parasite. Scale bar 1μm.
(TIF)

S1 Table Measurements in nm of conoid components, pre-conoidal rings and apical polar ring from Fig 1. For 2 of the 14 tomograms (number 3 and number 12) the ends of a few of the conoid fibres extended past the imaging volume, for these conoids, only the fibres where the start and end were visible were included in the analysis of fibre length, spacing, angle and curvature.
(XLSX)

S1 Movie Serial section cellular electron tomogram containing the apical complex of a freshly excysted sporozoite and segmentation of the dataset to illustrate the three-dimensional model. Conoid fibres (white); 2 pre-conoidal rings (PCR 1 and 2) (light blue and red), apical polar ring (gold) in association with sub-pellicular microtubules (green); rhoptry X 1 within the conoid (dark blue), micronemes X 2 modelled within the conoid (light green), microtubule-associated vesicle (yellow), intra-conoidal microtubule pair (pink);.
(M4V)

S2 Movie Serial section cellular electron tomogram of an infected MDKB cell 30 mins post-infection containing an electron lucent rhoptry and pore. Movie illustrates the tomogram data followed by the segmentation. Colour scheme as per S1 Movie figure legend.
(M4V)

S3 Movie A total of 25 sequential slices (~100nm thick) through an SBF-SEM dataset to illustrate a whole freshly excysted sporozoite used for quantitative analysis of organelles in Figs 4 and S4. (M4V)
