# Supplementary figures and images for: Cellular electron tomography of the apical complex in the apicomplexan parasite Eimeria tenella shows a highly organised gateway for regulated secretion
Source: PLoS Pathog. 2022 Jul 11;18(7):e1010666. doi: 10.1371/journal.ppat.1010666 (PMC9302750; doi:10.1371/journal.ppat.1010666)

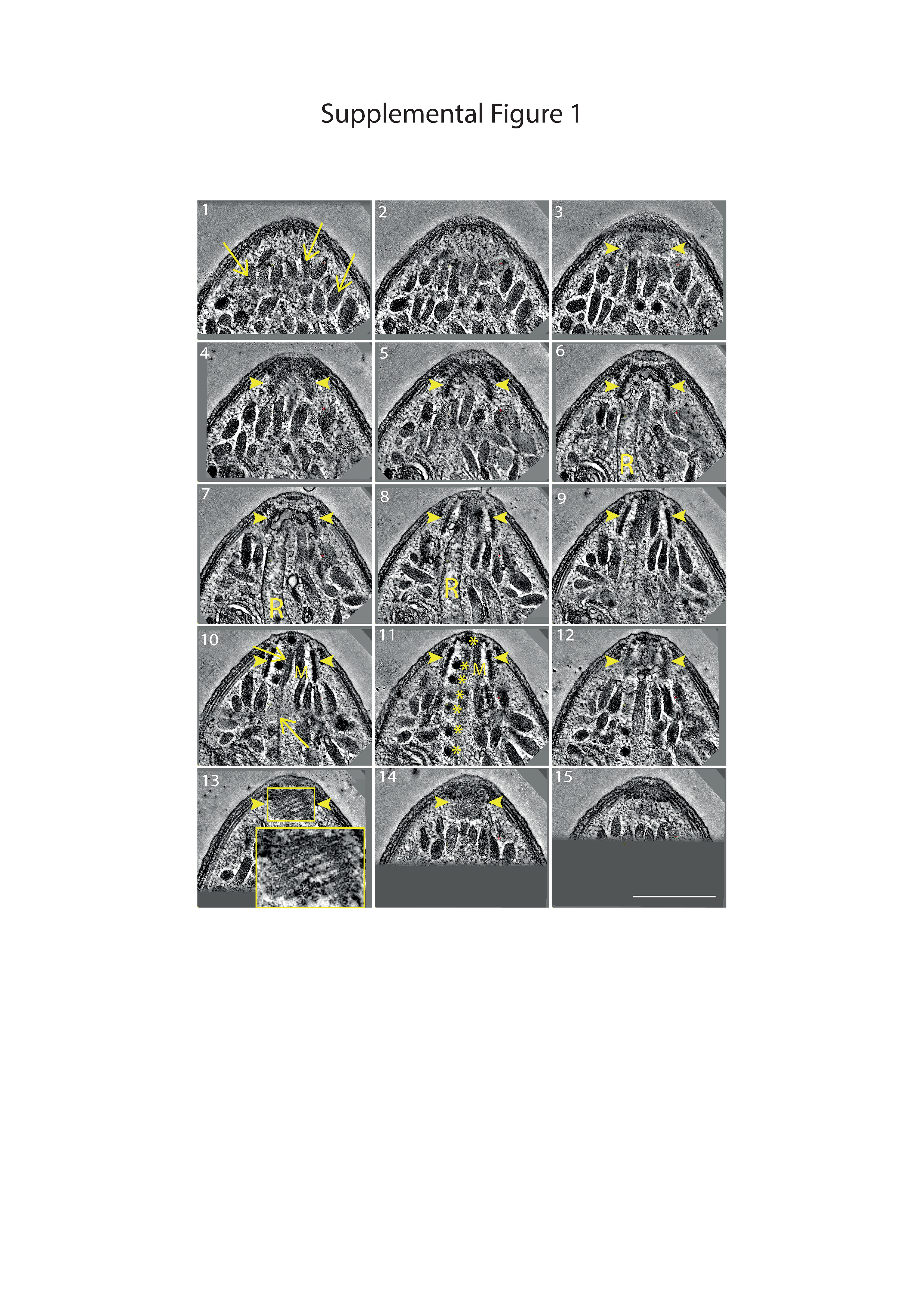

Supplement: S1 Fig — Scale bar—500nm. (TIF) [file ppat.1010666.s001.tif]

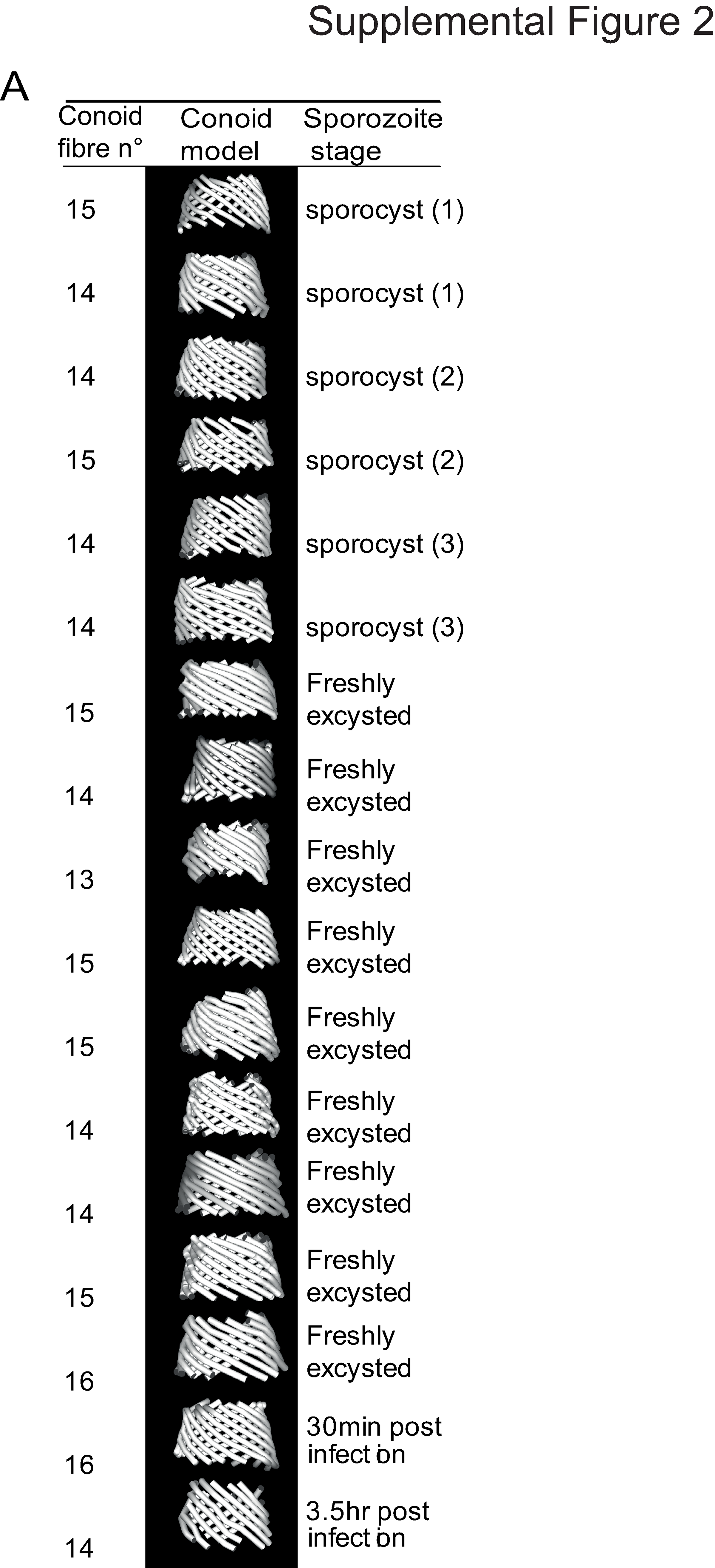

Supplement: S2 Fig — A: Segmentation of sporozoite conoids from pre-excystation sporozoites within sporocysts, freshly excysted and intracellular sporozoites. The numbers next to sporocysts indicates matching sporozoites within a sporocyst;. (TIF) [file ppat.1010666.s002.tif]

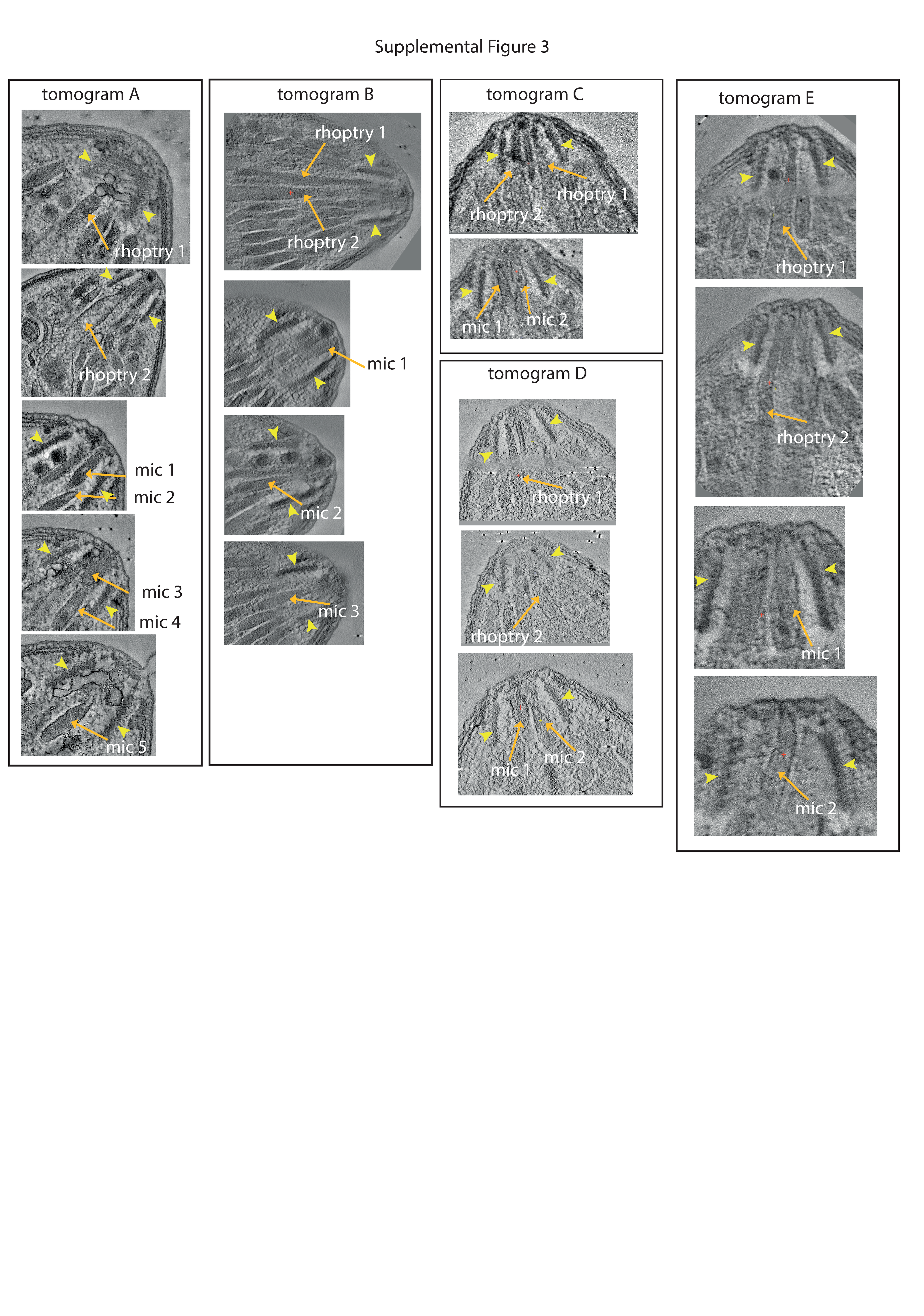

Supplement: S3 Fig — All micronemes that were either partially or fully within the conoid were included. Conoid is highlighted with yellow arrowheads in all examples. Tomogram A illustrates the presence of 2 rhoptries and 5 micronemes within the conoid. Mic 1 and 3 are closest to the plasma membrane overlying the conoid. Mic 2, 4 and 5 have partially entered the conoid area; Tomogram B, slices from a tomogram containing 2 rhoptries and 3 micronemes. Mic 1 is closest to the plasma membrane; Tomogram C shows slices from a tomogram with 2 rhoptries and 2 micronemes; Tomogram D illustrates slices from a tomogram containing 2 rhopries and 2 micronemes. Mic 1 is closest to the plasma membrane; Tomogram E, slices from a tomogram containing 2 rhoptries and 2 micronemes. (TIF) [file ppat.1010666.s003.tif]

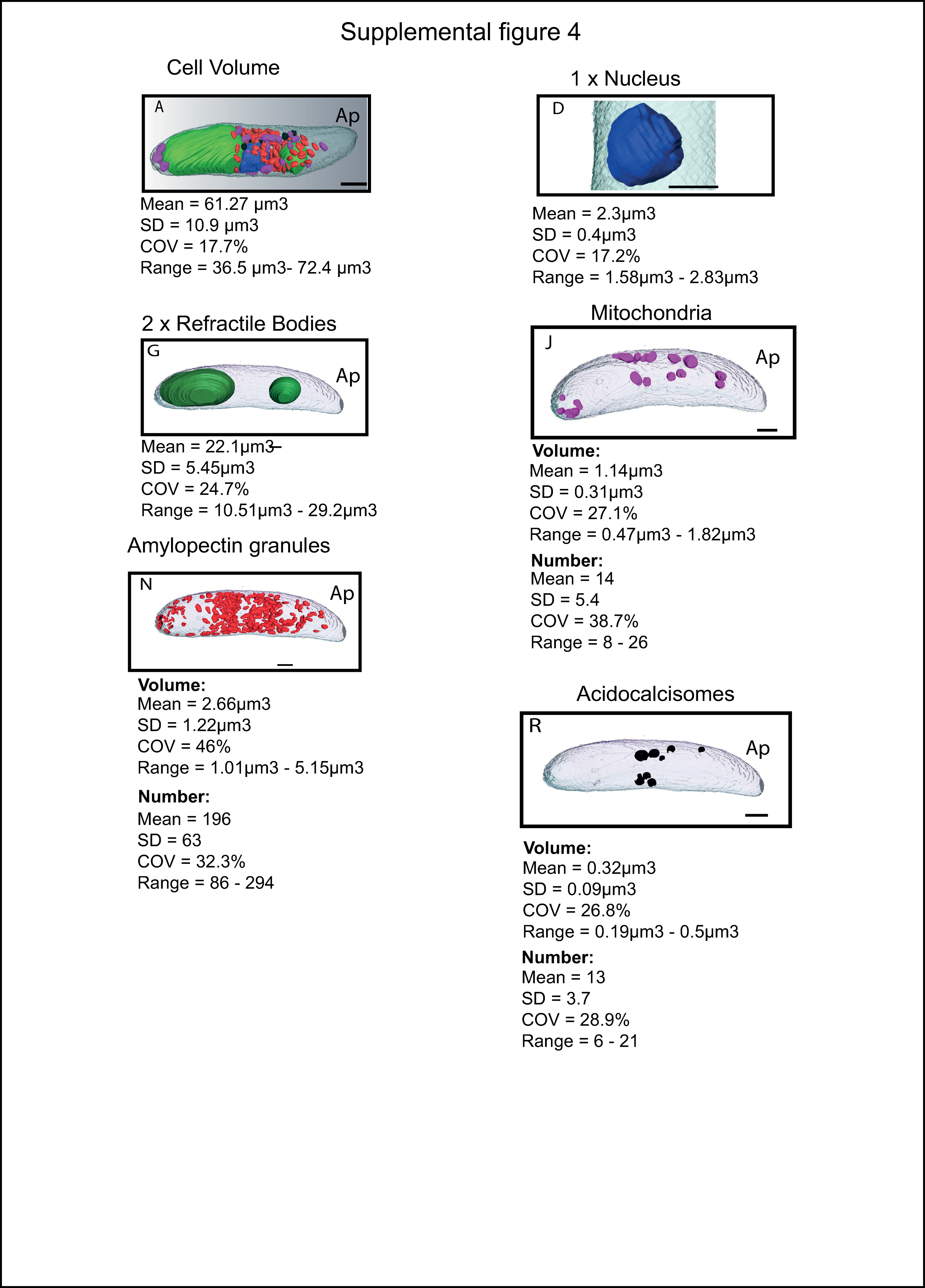

Supplement: S4 Fig — Analyses was calculated from segmented SBF-SEM data for each organelle in freshly excysted sporozoites. For each organelle the mean volume of an individual organelle is included, SD = standard deviation, COV = co-efficient of variation, range of volumes of a particular organelle or cell volume. The number of organelles per cell is included for amylopectin granules, acidocalcisomes and mitochondria. AP = Apical end of the parasite. Scale bar 1μm. (TIF) [file ppat.1010666.s004.tif]
